# Distress Screening among Patients with Hematological Malignancies: A Descriptive Cross-sectional Study

**DOI:** 10.31729/jnma.5194

**Published:** 2020-08-31

**Authors:** Bishal Paudel, Bishnu Dutta Paudel, Rupesh Mishra, Onika Karki, Rukmini Shahi, Bishesh Sharma Poudyal

**Affiliations:** 1 Department of Clinical Oncology, National Academy of Medical Sciences, Bir Hospital, Kathmandu, Nepal; 2 Clinical Hematology and Bone Marrow Transplant Unit, Civil Service Hospital, Minbhawan, Kathmandu, Nepal; 3 Department of Gyanecology and Obstetrics, Nepal Medical College, Jorpati, Kathmandu, Nepal

**Keywords:** *distress*, *distress thermometer*, *hematological malignancy*

## Abstract

**Introduction:**

Distress is a major concern during diagnosis and treatment of hematological malignancies. The Distress Thermometer is a commonly used screening tool to detect distress. The objectives of this study was to know the prevalence and identify distress score among patients with hematological malignancies in Nepal.

**Methods:**

A descriptive cross sectional study was carried out at the Hematology Unit of Civil Service Hospital after obtaining an ethical approval from the Institutional Review Committee (reference number 931/076/077). A convenient sampling technique was used for this study. Statistical Package for the Social Sciences version 20.0 was used. All patients within one week of diagnosis and before the start of definitive treatment of hematological malignancies were included in the study. National Comprehensive Cancer Network Psychosocial Distress Screening Tool was used to measure the seriousness of distress.

**Results:**

A total of 100 patients were enrolled in the study, among them 56 (56%) were male and 44 (44%) were female. The mean distress score in our study was found to be 5.68±1.75. Mean distress score among male and female patients were 5.84±1.65 and 5.48±1.86 respectively. Thirty three percentage (n=33) of patient had mild distress whereas, sixty six percentage (n=67) of patients experienced moderate to severe distress.

**Conclusions:**

There was a significant level of distress among the patients with hematological malignancies in Nepal. Therefore, distress screening should be done to all the patients when initial diagnosis is made.

## INTRODUCTION

Distress is a major concern at diagnosis and during the treatment of malignancy. The diagnosis of hematological malignancies creates immediate psychosocial distress for the patient and their family.^1^ The National Comprehensive Cancer Network (NCCN) defines distress in cancer as a “multifactorial unpleasant emotional experience of a psychological (cognitive, behavioral, emotional), social and/or spiritual nature that may interfere with the ability to cope effectively with cancer, its physical symptoms and its treatment”.^2^

The Distress Thermometer (DT) is a commonly used screening tool to detect distress in cancer patients.^3^

Various studies reported a rising incidence of distress among patients with a variety of cancers including hematological malignancies.^4–6^ Distress is underrecognized in cancer treatments in Nepal. Distress should be assessed and managed in all patients with hematological malignancies.

The objectives of this study was to know the prevalence and identify distress score among patients with hematological malignancies in Nepal.

## METHODS

A Descriptive cross sectional study was carried out at Hematology Unit of Civil Service Hospital after study proposal was accepted by IRB, NAMS from January 2020 to May 2020. All patients within one week of diagnosis and before the start of definitive treatment of hematological malignancies were included in the study. Those with pre-existing psychiatric illness and refused to enrol were excluded from the study. Eligibility criteria were judged during the initial clinical evaluation of the patient. An informed written consent was obtained. Demographic data of patients were collected. Standardized Performa were used to record the data in accordance with the protocol's instructions. The sample size was calculated using the following formula.

n= Z^2^ × p × (1-p)/e^2^

where,

n = Sample size
Z = Statistic for level of confidence
P = Expected prevalence
e = Margin of error
Here, Z = 95 % CI = 1.96
P = 45.5% =0.455
e = 10%=0.1
n = 1.96^2^ × 0.455 (1-0.455)/0.1^2^ = 95.26
Hence, Sample size was 96.

NCCN's Psychosocial Distress Screening Tool was used to measure seriousness of distress. Relevant variables for each individual included age, diagnosis, gender, marital status were collected. The investigators asked the patients to choose a number from 0 to 10 that reflects how much distress one felt over the past week. Ten is the highest level of distress one can imagine, and 0 is no distress. If response was 5 or above, it was labelled as a moderate-to-high degree of distress.

Data was entered and exported to statistical analysis system program (SPSS) version 20. Simple frequencies, percentages, measures of central tendency and variability were calculated.

## RESULTS

A total of 100 patients were enrolled in the study, among them 56 (56%) were male and 44 (44%) were female. The mean age group in our study was 43±16 years. Thirty three percentage (n = 33) of patients had mild distress out of which 20 (20%) were female and 13 (13%) were male. Sixty seven percentage (n = 67) had moderate to severe distress out of which 24 (24%) were female and 43 (43%) were male. The mean distress score in our study was found to be 5.68±1.75, and distress score among male and female patients were 5.84±1.65 and 5.48±1.86 respectively (Table 1). Younger patients were found to be more distressed than older patients. The mean distress score was 4.88±1.45 in patients above 60 years and it was 6.50±1.68 in 21-40 years (Table 2).

**Table 1:** Severity of distress according to gender.

Gender	Mild	Moderate/Severe	Grand Total
Female	20.00%	24.00%	44.00%
Male	13.00%	43.00%	56.00%
Grand Total	33.00%	67.00%	100.00%

**Table 2:** Distress score by age and gender.

Age group	Female	Male	Grand Total
<20	5.80±2.59	6.63±1.30	6.31 ±1.84
21-40	6.60±1.64	6.36±1.80	6.50±1.68
41-60	4.77±1.64	5.55±1.63	5.32±1.65
>61	4.64±1.36	5.33±1.63	4.88±1.45
Grand Total	5.48±1.86	5.84±1.65	5.68±1.75

A total of 39 patients (39%) were diagnosed with acute leukemia, among them 9 (9%) had mild distress and 30 (30%) had moderate to severe distress. There were 24 patients (24%) diagnosed with lymphoma, out of which 11 (11%) had mild distress and 13 (13%) had moderate to severe distress. Myelodysplastic syndrome was diagnosed in 11 (11%) of patients, out of which 5 (5%) had mild and 6 (6%) had moderate to severe distress.

Similarly, 13 (13%) were diagnosed with multiple myeloma, among them 5 (5%) had mild distress and 8 (8%) had moderate to severe distress (Table 3). Likewise, 13 (13%) of the study group had myeloproliferative neoplasm out of which 4 (4%) had mild distress and remaining 9 (9%) had moderate to severe distress. The mean distress score was found to be 6.33±1.84, 5.29±1.68, 5.18±1.89, 5.54±1.61 and 5±1 in acute leukemia, lymphoma, myelodysplastic syndrome, multiple myeloma and myeloproliferative neoplasm respectively.

**Table 3:** Severity of distress according to disease types.

Diagnosis	Mild	Moderate/Severe	Grand Total
Acute Leukemia	9.00%	30.00%	39.00%
Lymphoma	11.00%	13.00%	24.00%
Myelodysplastic syndrome	5.00%	6.00%	11.00%
Multiple myeloma	4.00%	9.00%	13.00%
Myeloproliferative neoplasm	4.00%	9.00%	13.00%
Grand Total	33.00%	67.00%	100.00%

**Table 4:** Distress score by disease type.

Diagnosis	Mean Distress Score
Acute Leukemia	6.33±1.84
Lymphoma	5.29±1.68
Myelodysplastic syndrome	5.18±1.89
Multiple myeloma	5.54±1.61
Myeloproliferative neoplasm	5.00±1.00

## DISCUSSION

Distress is common among hematological malignancies. The NCCN standard of care guidelines for the clinical setting state that “all patients should be screened for distress at their initial visit, at appropriate intervals, and as clinically indicated especially with changes in disease status”. The recommended assessment tool is the Distress Thermometer, which measures global distress via a numerical rating scale.^2^ Therefore, Distress Thermometer was used to measure the Distress score in our study in the initial visit.

A score of 5 or greater on the thermometer trigger further evaluation and should be referred to a psychosocial service.^7^ The mean distress score in our study was found to be 5.68±1.75. Therefore, all the patients whose distress score was more than five in our study were sent for psychosocial counselling.

In our study distress was found to be prevalent in all the patients irrespective of disease types. However, the distress score was different among disease subtypes.

A study done by Albrecht TA et.al, reported that in patients diagnosed with acute leukemia, distress has been found in about 45.5% of patients.^8^ In our study 39% patients were acute leukemia out of which 9% had mild distress and 30% had mod to severe distress. A study done by V Manitta et.al reported that the psychosocial distress was higher among the patients with advanced disease stage of haematological malignancies.^5^ In our study, the mean distress score was higher in patients with acute leukemia and was low in myeloproliferative neoplasm. The high distress Score in acute leukemia patients may be because of the poor prognosis of disease, longer treatment duration, higher economic burden and prolonged hospital stay.

There were no gender differences in anxiety or overall distress severity.^9^ Likewise, our study also confirmed no gender difference in distress score. Those who are younger tend to be more prone to distress than older ones.^10^ Emotional distress was inversely related to age.^11^ Our study also showed older patients were less distressed than younger patients.

Distress is associated with increased symptoms and length of hospitalization, as well a reduced health-related quality of life (HQoL) and may lead to modifications in treatment regimens.^12^ Therefore, early recognition of distress is crucial and distress screening should be incorporated in the treatment protocol of hematological malignancies.

## CONCLUSIONS

Patients with hematological malignancies are at a high risk of experiencing cancer-related distress in Nepal. Distress Thermometer can be used as a short screening tool. Young patients with haematological malignancies and patients with acute leukemias were found to be more distressed. Hence apart from usual treatment, psychosocial counseling to address distress should be incorporated in the management of patients with hematological malignancies.
